# Sudden Cardiac Risk Stratification with Electrocardiographic Indices - A Review on Computational Processing, Technology Transfer, and Scientific Evidence

**DOI:** 10.3389/fphys.2016.00082

**Published:** 2016-03-07

**Authors:** Francisco J. Gimeno-Blanes, Manuel Blanco-Velasco, Óscar Barquero-Pérez, Arcadi García-Alberola, José L. Rojo-Álvarez

**Affiliations:** ^1^Department of Signal Theory and Communications, Miguel Hernández UniversityElche, Spain; ^2^Department of Signal Theory and Communications, University of de AlcaláAlcalá de Henares, Spain; ^3^Department of Signal Theory and Communications, Rey Juan Carlos UniversityFuenlabrada, Spain; ^4^Arrhythmia Unit, Hospital Universitario Virgen de la ArrixacaEl Palmar, Spain

**Keywords:** sudden cardiac death, risk stratification, computational algorithms, scientific evidence, technology transfer, heart rate variability, heart rate turbulence, T–wave alternans

## Abstract

Great effort has been devoted in recent years to the development of sudden cardiac risk predictors as a function of electric cardiac signals, mainly obtained from the electrocardiogram (ECG) analysis. But these prediction techniques are still seldom used in clinical practice, partly due to its limited diagnostic accuracy and to the lack of consensus about the appropriate computational signal processing implementation. This paper addresses a three-fold approach, based on ECG indices, to structure this review on sudden cardiac risk stratification. First, throughout the computational techniques that had been widely proposed for obtaining these indices in technical literature. Second, over the scientific evidence, that although is supported by observational clinical studies, they are not always representative enough. And third, via the limited technology transfer of academy-accepted algorithms, requiring further meditation for future systems. We focus on three families of ECG derived indices which are tackled from the aforementioned viewpoints, namely, heart rate turbulence (HRT), heart rate variability (HRV), and T-wave alternans. In terms of computational algorithms, we still need clearer scientific evidence, standardizing, and benchmarking, siting on advanced algorithms applied over large and representative datasets. New scenarios like electronic health recordings, big data, long-term monitoring, and cloud databases, will eventually open new frameworks to foresee suitable new paradigms in the near future.

## 1. Introduction

Sudden Cardiac Death (SCD) describes the unexpected natural death from a cardiac cause within a short period of time (generally ≤ 1 h from the onset of symptoms if witnessed, or within 24 h of having been observed alive if unwitnessed), in a person without any prior condition that would appear fatal (Zipes and Wellens, 1998; Priori et al., 2001; Organization, 2005). The definition for sudden death is similar to SCD, except for its origin is from any cause. Nevertheless, the main cause of sudden death is cardiac origin, and SCD is mainly due to: (1) arrhythmic causes, such as ventricular tachycardia (VT), ventricular fibrillation (VF), or asystole; (2) other structural heart disease causes, such as congenital heart disease; or (3) abnormal functioning of the autonomic nervous system, which is not a death cause itself, but it can promote causes such as arrhythmic or hypertension death (Pratt et al., 1996; Priori et al., 2001; Zipes et al., 2006). The SCD mechanism in the last cases is usually VT or VF.

SCD remains a major cause of mortality in industrialized countries, and in order to reduce its incidence, methods allowing accurate patient stratification in terms of risk have been intensely scrutinized and developed. Whereas, a number of studies suggest that most SCD episodes are given in patients with coronary disease or cardiomyopathy, episodes can also occur in people without previous symptoms or signs of heart disease, and regrettably, there is no accurate enough method to effectively predict SCD in these conditions. Risk stratification of SCD in patients differs substantially depending on the underlying cardiopathic basis, since the prognostic significance of noninvasive studies and efficacy of therapeutic measures are strongly etiology-dependent (Villacastín et al., 2004).

While the measurement of left ventricular ejection fraction (LVEF) is widely used as the gold standard for detecting SCD high-risk patients, other noninvasive techniques and measurements have been proposed, such as late potentials, heart rate variability (HRV), heart rate turbulence (HRT), T–wave alternans (TWA), or deceleration capacity. In recent years, an intense research has been driven for the development of SCD risk predictors as a function of computational indices obtained from the analysis of the electrocardiogram (ECG). However, these techniques are not currently used in the clinical routine. A surprising fact is the lack of consensus about the most adequate computational methods for preprocessing or signal conditioning in order to extract the clinically relevant information (Antezano and Hong, 2003).

However, the interest of this subject has generated a number of reviews, tutorials, and guidelines on the clinical fundamentals and scope of SCD (Zipes and Wellens, 1998; Priori et al., 2001; Zipes et al., 2006; Goldberger et al., 2008). In contrast to them, this work is intended to illustrate the current situation in the field of SCD risk stratification with computerized indices from a practical standpoint. For this purpose, we address this study from a three-fold perspective: (a) the role of computational processing techniques and its diversity; (b) the current technology transfer and its limitations; and (c) the need for scientific evidence and its precedents.

Three relevant SCD computational markers are discussed in detail, which have been chosen for their widespread relevance in the field. HRT is assessed with an easy and clear methodology with very few variations from the initially proposed one, hence it represents a well established and standardized prediction technique. Conversely, HRV has been extensively studied and measured throughout a huge number of different proposed techniques and indices. Finally, TWA is recently gaining much clinical interest, but again, plenty of methods have been proposed for its computation, which makes unclear the consistency between clinical and technical literature.

The outline of this review is as follows. For better understanding the computational issues of the topic, Section 2 addresses the general background on the SCD origin, and Section 3 gives an overview on the used and proposed testing methods in the clinical practice. Then, in Section 4, three representative families of ECG derived indices are explored with focus in the algorithmic implementations, namely, HRT, HRV, and TWA. Section 5 summarizes the technology transfer by analyzing the algorithms implemented in the commercial equipments and the patents in the field, whereas Section 6 conveys a clinical and scientific evidence landscape. Section 7 contains the discussion and conclusions of the present work.

## 2. SCD origin

It is fundamental to understand that the SCD risk stratification methods provide us with information related to the several physiological aspects that may be affected during the course of an SCD episode. The facts about SCD that are currently established and accepted are next summarized.

First, most sudden death cases have usually cardiac origin, and they mostly arise from some arrhythmic mechanisms in malignant ventricular arrhythmia. These arrhythmias are more frequently found in cardiopathies with extensive structural affection of the ventricular myocardium (such as large myocardial infarction and other kinds of cardiomyopathy), and in patients with genetic abnormalities in the ionic channels (such as long QT and Brugada syndrome channelopathies). In most cases, the mechanism that elicits terminal arrhythmias is considered to be a reentry in its multiple forms, depending on the clinical scenario, such as anatomical or functional barriers, and rotors. Second, non-sustained arrhythmias (basically ventricular extrasystoles) may operate as triggers for the reentry development in some cases, because the premature beat is spread throughout a heterogeneous substrate, thus generating a non–homogeneous slow conduction that allows the reentry. And third, it is also known that in many of these cases, the autonomous system, which innervates the heart and modifies the properties of the ionic channels, can play a modulating role that may allow the appearance or maintenance of the aforementioned reentries or their triggering causes (Goldberger et al., 2008; Priori et al., 2015).

Practical SCD computational predictors should be capable of identifying patients with either substrate, or triggers, or modulators, that lead to deathly arrhythmias, namely, non-tolerated VT and VF. Accordingly, they should provide information about the features described next.

1. *The presence of a substrate allowing reentries*, which includes the detection of slow conduction pathways in the myocardium, and it is given by increased QRS duration, late potentials (including early and afterdepolarizations), left bundle-branch block, or specific ECG patterns (e.g., Brugada), among other possible factors. In this setting, the demonstration of arrhythmia inductibility by maintaining a reentry in an electrophysiological study (EPS) shows the existence of a pro-arrhythmic substrate. The presence of anatomic barriers, heterogeneity, and myocardial conduction dispersion (e.g., due to scars or fibrosis) are also substrate indicators. Finally, the spatial or temporal repolarization dispersion, which makes possible reentries, can be detected, for instance in the QT dispersion or in the TWA presence.
2. *The existence of triggers*, which can be from extrasystole and non-sustained VT, to the well-known R on T phenomenon, among others (Engel et al., 1978).
3. *The existence of modulator abnormalities*, which is mostly due to three physiological alterations. First, the *autonomous nervous system alterations* on the sinus node control, which are observed via HRV, HRT, and on baroreflex sensitivity (to be described next). Note that the presence of alterations in these indices is not itself a mechanism of SCD related arrhythmias, but instead it represents a way of evaluating the function of the cardiovascular autonomous nervous system, in such a way that abnormal function is assumed to promote ventricular arrhythmias that are the actual cause of SCD. Second, the *performance alteration of ionic channels*, such as mutations with high arrhythmic risk. And third, many *other modulating factors*, including the ischemia, the celular hydrolytic environment, or the presence of drugs modifying the electric properties of the cells.

## 3. Testing methods

According to the testing method required to obtain the information, the existing SCD risk stratification techniques can be divided into invasive and noninvasive ones. The former mostly consists of EPS, and the later include 12-leads ECG, medical image techniques, Holter ECG, high-resolution ECG, and baroreflex test. Other testing methods, such as genotype determination or stress test, can be useful in patients with some specific cardiopathies causes (Goldberger et al., 2008; Priori et al., 2015).

### 3.1. Information from the EPS in the patient

The EPS uses a set of intracardiac catheters, guided by X-ray or other systems, in order to register and record the internal cardioelectric activity. The EPS-based SCD risk stratification mainly consists of trying to induce ventricular arrhythmias (VT or VF) by using electric extra-stimuli according to an adequate programmed interval sequence in the ventricles, and for this purpose it should not be longer than 20 or 30 min. The EPS is not useful for the diagnosis of long QT syndrome or hypertrofic myocardiopathy, and it does not provide either with relevant risk information in these pathologies.

The usefulness of EPS for SCD prediction has been only validated in ischemic cardiopathy after myocardial infarction, and weakly validated in Brugada syndrome channelopathy and in some other cardiopathies. For other cardiopathies, either there are not enough data, or it is well known that EPS does not provide with a useful SCD risk stratification criterion. Nevertheless, the usefulness of EPS in ischemic cardiopathy was validated in old studies with limitations, hence they are seldom used in today clinical practice, except for very specific situations, e.g., in patients with non-filiated origin syncope, old infarction, and reasonably preserved ejection fraction. Note also that patients with severe ventricular disfunction have straightforward indication for Implantable Cardioverter Defibrillator (ICD), hence they are not referred to EPS.

In addition, EPS has not been found to be very useful with ventricular programmed stimulation for SCD risk stratification in dilated myocardiopathy, as far as it is Class IIb in current clinical practice guidelines (usefulness/efficacy is less well established by evidence/opinion, may be considered), and it is based in a meta-analysis (Goldberger et al., 2014; Priori et al., 2015). Neither it has in hypertrophic myocardiopaty, for being Class III in current guidelines (invasive EPS with PVS is not recommended for stratification of SCD), and for being something not much discussed except for old publications. Finally, neither it has in right ventricular arrhythmogenic dysplasia, for being Class IIb in the same guidelines (it can be considered for sudden death stratification, but with low basement), only for being based in retrospective studies in which ventricular arrhythmia induction is a significant predictor in multivariate analysis (Roguin et al., 2004; Bhonsale et al., 2011), but no prospective studies have been designed for clarifying this, hence its robustness is low.

### 3.2. Conventional ECG

The conventional ECG is a well-known graphical representation of the cardiac electrical activity. Among the number of ECG standards depending on the followed objective, the most commonly used is the 12-lead one. The basis of this system is to evaluate a set of weighted potential differences between specific sites of the body surface where electrodes are placed. Every ECG is characterized by the cyclical occurrence of time-varying patterns with different frequency content, defining specific and known shapes that are related to relevant and macroscopic noticeable electric activity of the heart. The most relevant waves over the beat cycle are the P wave (atrial depolarization), followed by the QRS complex (ventricular depolarization), and covering up to the T wave (ventricular repolarization). The ECG is a quite standard inexpensive non-invasive medical procedure with immediate results, and available in all medical environments, and for this reason, it plays a significant role for cardiovascular diseases screening and diagnosis, metabolic disorders detection, and SCD predisposition.

Accordingly, the ECG can be helpful for diagnosis of the underlying cardiopathy, but it also can show elements suggestion increased SCD risk, for instance, a Type I pattern in Brugada syndrome, an elongated QT interval in the long-QT syndrome, or a bundle-branch block in ischemich cardiopathy and in miocardiopathies.

The QRS duration is a manifestation of the existence of a conduction delay or intraventricular blocking. The duration of ventricular activation is usually measured by a 12-lead ECG, and under normal conditions it is lower than 120 ms. The QRS duration is a highly reproducible measurement, with less than 5% variation. A moderate amount of data shows that increased QRS duration identifies patients at increased risk of SCD, although the data are not uniform. In the absence of prospective studies specifically designed to address this problem, the use of QRS duration for risk stratification of SCD in patients with CHF is not recommended at present (Goldberger et al., 2008). Recent studies have demonstrated that QRS duration is an independent predictor of SCD risk, though they refer to general population, rather than cardiopathy (Kurl et al., 2012; Laukkanen et al., 2014).

The QT interval is a reflection of the combination of the durations of the ventricular action potentials. Its value decreases with increasing heart rate, and several correction formulas have been suggested for its correction according to this factor, being the Bazett's equation (QT interval divided by the squared root of RR interval) one of the most used ones (Ahnve, 1985). The normal corrected QT interval is slightly shorter in men than in women. The QT interval measurements have been shown to be highly reproducible, but the need for certain correction formulas and the use of different corrections throughout studies limits their populational comparison.

### 3.3. Noninvasive medical image techniques

A well established relationship exists between left ventricular systolic dysfunction and death from progressive heart failure (HF) and ventricular arrhythmias in patients who have suffered an acute myocardial infarction (AMI). The LVEF reduction has been the most consistently reported SCD risk factor in HF patients, and an important predictor of cardiac (and sudden) mortality in the long term after AMI. A LVEF ≤ 30 or 40% is used as the threshold for identifying high-risk individuals. At present, LVEF is considered a limited predictor, and is often impossible to distinguish between patients with high arrhythmic mortality and those with a high mortality due to pump failure (Villacastín et al., 2004; Goldberger et al., 2008). Therefore, the limited sensitivity of this test makes necessary its combination with other diagnostic tests.

In addition to helping to the diagnosis of the underlying cardiopathy, several cardiac image techniques also can provide with SCD risk indicators. In this setting, they are used to estimate the LVEF for SCD risk stratification, which can be estimated by means of echocardiography, isotope ventriclegraphy, cardiac computerized axial tomography, cardiac nuclear magnetic resonance, or conventional contrast ventriclegraphy. The mostly widespread used technique is echocardiography, for its ease, lower price, and absence of irradiation.

The depressed LVEF is a significant risk factor in ischemic cardiopathy, in myocardiopathies, in severe septal hypertrophy, in hypertrophic myocardiopathy, among others. Also, cardiac nuclear magnetic resonance can be used to estimate the degree of myocardial fibrosis, which has been related to the ventricular arrhythmia episodes in some cardiopathies.

### 3.4. Long-term ECG monitoring

The ambulatory electrocardiography, or Holter, is a diagnostic method consisting of ECG recordings from 24 to 48 h in two or three chest leads (some current systems provide with 12 leads recording). These recordings are subsequently analyzed by using a computer, and relevant electrophysiological events can be easily detected, including several potential and proposed markers in SCD risk stratification, such as ventricular extrasystoles, non-sustained (NS) VT, or VT episodes, as well as TWA, HRT, or HRV indices, whose alterations can promote ventricular arrhythmias.

There are a number of arrhythmic events associated to SCD risk that are mostly noticeable on Holter ECG. Particular attention has been paid to Ventricular Premature Beats (VPB), with occurrence of 70–95% of HF patients (Teerlink et al., 2000; Villacastín et al., 2004). Also, left bundle-branch block is an arrhythmic event which, in turn, has been analyzed in the literature as a specific marker of SCD (Baldasseroni et al., 2002; Iuliano et al., 2002), although findings could not be considered very conclusive from a prognostic perspective (Villacastín et al., 2004). NSVT has also been observed on 50–80% of the patients with HF and cardiomyopathy (Teerlink et al., 2000), and previously observed NSVT is usually a significant predictor in univariate analysis, but it does not remain as an independent predictor in multivariate analysis (Villacastín et al., 2004). Another remarkable arrhythmic event is atrial fibrillation (AF), which is caused by chaotic electrical activity at the atrium, resulting in an irregular ventricular response and minimum oscillations of the baseline or f-waves (Villacastín et al., 2004).

### 3.5. Other tests

Other relevant tests that have been paid relevant attention in the context of SCD risk stratification are the following (see Villacastín et al., 2004; Goldberger et al., 2008 and references therein).

*ECG Signal Averaging Techniques* detect the presence of arrhythmic substrate in terms of slow conduction paths. In particular, ventricular late potentials are high frequency potentials with small amplitudes found in the final portion of QRS complex and the onset of the ST segment. These potentials are associated to depolarization of slow-conduction areas at the edges of the scarred myocardium. Delayed conduction and unidirectional block tend to favor reentry, which is believed to be the cause of certain VT. However, signal averaged ECG is not currently used for risk prediction of SCD. It was formerly used to predict the inductibility of ventricular arrhythmias during EPS, which is indirectly related to SCD, but published data are from several decades ago, and they are not easily applicable to current populations and therapies. Nowadays, the only clinical indication for this technique is helping to the diagnosis of right ventricular arrhythmogenic dysplasia in some cases, and even in this pathology it has not been shown to have SCD predictive capacity.

*Baroreflex Sensitivity* refers to the heart response, in terms of RR intervals, to changes in blood pressure. Baroreflex mechanisms have been established as central element to the regulation of the cardiovascular system. It is usually measured with a polygraph or conventional electrocardiograph system during a procedure in which arterial pressure is pharmacologically modified, hence allowing to detect alterations in the autonomous function.

Studies examining the vagal reflex in patients with recent HF provide prognostic information independent of the LVEF and additional to the information provided by HRV measurements. The main restriction of this method is the requirement to simultaneously record blood pressure and HRV signals, which is not usual in the clinical practice. New measurements have been developed in recent years, such as baroreflex sensitivity turbulence in heart rate, which reflects the fluctuation of the cardiac cycle length in sinus rhythm after an isolated PVB.

## 4. Algorithmic and computational indices

This section summarizes the main computational and algorithmic implementations that have been used in the three families of SCD risk stratification indices.

### 4.1. HRT indices

**Figure 1:**
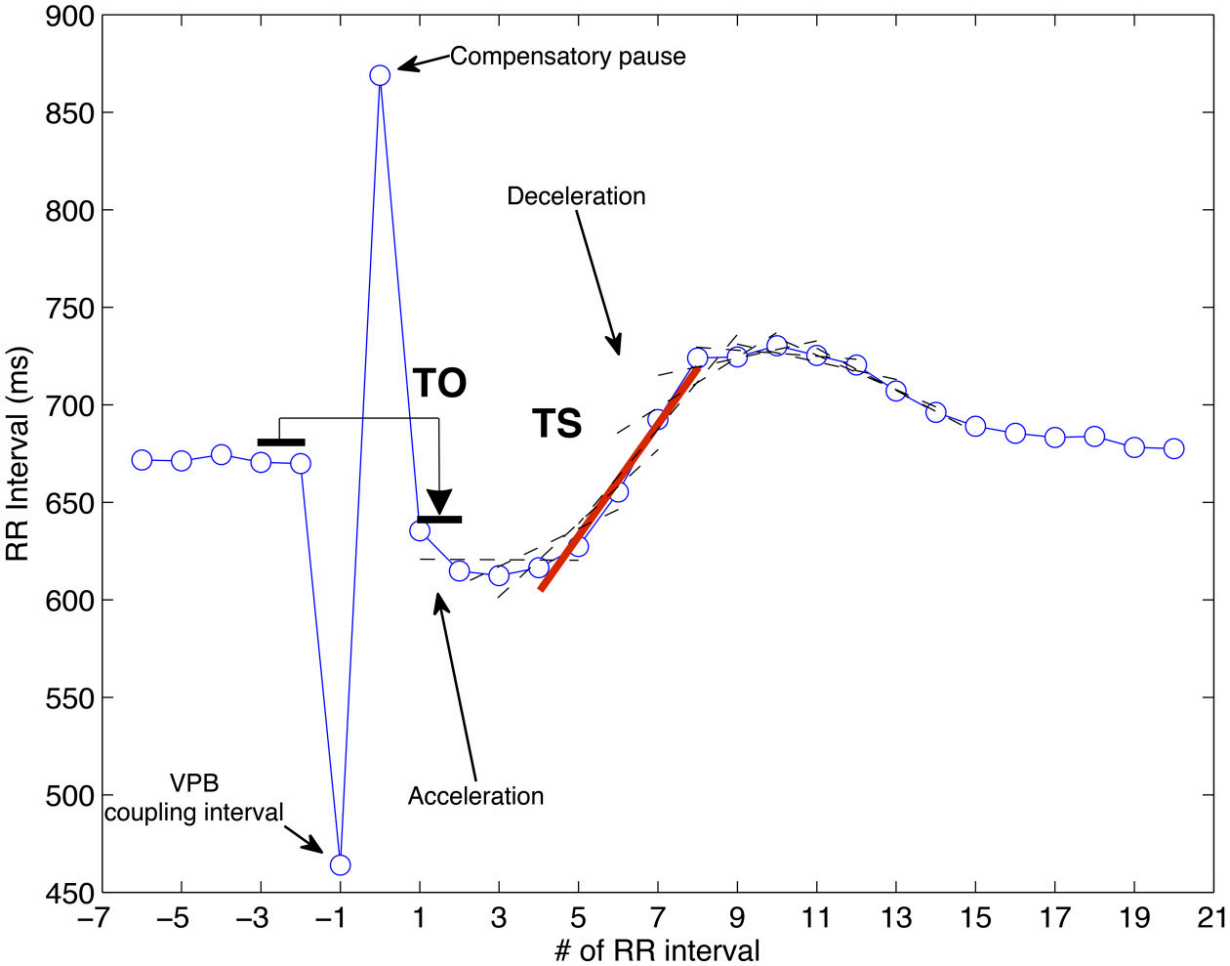
**Biphasic response after a VPB in an RR interval time series (circles)**. The computation of both TO and TS is also represented.

HRT describes the short–term fluctuation in ECG cycle length that follows a VPB. The turbulence can be very well identified in RR interval time series and its regular pattern exhibits an initial sinus rhythm acceleration after the VPB, followed by a subsequent deceleration to finally return to pre–ectopic levels (Watanabe and Schmidt, 2004; Bauer et al., 2008). Figure 1 depicts a typical averaged tachogram over which the turbulence phenomenon is observed. It comprises the VPB surrounded by several sinus RR intervals, usually at least two before the coupling interval and 15 after the compensatory pause.

The physiological mechanism of HRT is likely based on a baroreflex source in such a way that changes in blood pressure are also manifested in the heart rate (HR). The lower ventricular filling and ineffective contraction due to a VPB produces a reduced pressure, which causes vagal inhibition and increases the HR. Subsequently, this heart rhythm growth induces a post–compensatory pause increased pressure resulting in vagal activity restitution, which in turn leads to sinus deceleration. Any deviation from this pattern may reflect anomalous autonomic function, thus, patients at risk show an attenuated or even entirely missing HRT, and this difference on the HRT response has been proven to be an informative predictor of mortality and SCD (Schmidt et al., 1999; Barthel et al., 2003).

The measurement of HRT is carried out by means of two parameters, namely, Turbulence Onset (TO) and Turbulence Slope (TS), which quantify the two phases described above. The early acceleration is characterized by

(1) $$\begin{array}{l} TO=\frac{\left(RR_{1}+RR_{2}\right)-\left(RR_{-3}+RR_{-2}\right)}{\left(RR_{-3}+RR_{-2}\right)}\cdot 100 \end{array}$$

where *RR*_−3_ and *RR*_−2_ are the two RR intervals preceding the coupling interval, while *RR*_1_ and *RR*_2_ are the two RR intervals immediately following the compensatory pause. The spots used for calculating TO are identified in Figure 1, which for this case clearly reflects rhythm acceleration corresponding to a negative value (healthy response) when computed as in Equation (1). On the other hand, the sinus deceleration, attributed to the second turbulence phase, is quantified by TS, which is the slope of the steepest regression line observed over any sequence of five consecutive RR intervals starting within the first 15 sinus rhythm RR intervals after the compensatory pause. The thick line superimposed over the deceleration portion of the tachogram observed in Figure 1 is the one reporting the steepest slope from which TS is determined. In most clinical studies, the values *T*0 < 0 and *TS* > 2.5 ms/RR interval are considered as normal. There are three categories for SCD risk stratification based on HRT indices, namely, Category 0 (both TO and TS are normal), Category 1 (either TO or TS is abnormal), and Category 2 (both are abnormal). Category 0 also comprises the case when HRT cannot be calculated due to unsuitable tachogram.

In analyzing turbulence from a single VPB, background effects and noise related to HRV severely affects any individual tachogram, so in practice, the aforementioned parameters are assessed over the mean of a number of selected VPB taken from a long-term Holter. Thus, the shape shown in Figure 1 is a clean VPB waveform which corresponds to an averaged tachogram. For reliable construction, at least five individual tachograms need to be averaged, but normally, tachograms from all VPBs on a Holter recording are used. At the same time, not all the VPB tachograms are suitable, hence, those that do not hold some specific morphological conditions are considered useless and invalid for averaging (Watanabe and Schmidt, 2004; Bauer et al., 2008).

Intra-patient long-term averaging of VPBs in order to reduce the noise level of the HRT signal provides only long-term HRT computational indices. In Rojo-Álvarez et al. (2009), a method for denoising individual VPBs was proposed using Support Vector Machines (SVM) for regression, by using HRT stimulated during EPS as a low-noise gold standard. This approach provided with HRT measurements in a 24–h Holter patient database with significant reduction in the bias and the variance. In this setting, HRT is well-known to be affected by several physiological factors, mainly heart rate and coupling interval of the VPB. Despite the physiological hypothesis to explain the HRT as a baroreflex response after the VPB, several studies have shown different results about the relationship between coupling and HRT parameters, sometimes with results apparently opposite to the hypothesis of HRT baroreflex source (Watanabe, 2003; Savelieva et al., 2003; Lee et al., 2004; Schwab et al., 2004). In Barquero-Pérez et al. (2012), a nonlinear regression model was used to assess the influence of coupling interval and heart rate on HRT, using the same data as in Rojo-Álvarez et al. (2009) from EPS and from Holter recordings. Results showed that the non-averaged tachogram analysis with the nonlinear regression model is able to explain the influence of the coupling interval on the HRT for healthy patients in accordance with the baroreflex hypothesis.

Both TS and TO, assessed over averaged tachograms, are so far the widely accepted turbulence measures for risk stratification. Nonetheless, although several other HRT measures have been proposed, they have scarcely been applied in clinical studies. Regarding TS modifications, a new figure independent of heart rate and the number of VPBs was given in Hallstrom et al. (2004), and a corrected TS normalized with respect to the systolic and diastolic blood pressure was used in Malberg et al. (2004). Furthermore, new parameters (such as turbulence dynamics, turbulence frequency decrease, turbulence timing, turbulence jump, and correlation coefficient of TS) have been presented, though their risk stratification capabilities have not yet been fully demonstrated (Watanabe, 2003).

Finally, an alternative approach to quantify HRT was proposed in (Solem et al., 2008; Martínez et al., 2010; Smith et al., 2010), from which a statistic test is performed over the turbulence waveform, modeled as the sum of HRT and HRV, and where the HRV is taken here as the noise source. The method relies on an extension of the integral pulse frequency model, defined for describing rate variability, where the turbulence phenomenon is included. Thus, the turbulence is treated as a function of time, instead of a tachogram, over which the generalized likelihood ratio test is applied. The method exhibits good performance on simulated data as well as in a limited set of ambulatory recordings, but it still needs further research on representative clinical studies.

### 4.2. HRV indices

**Figure 2:**
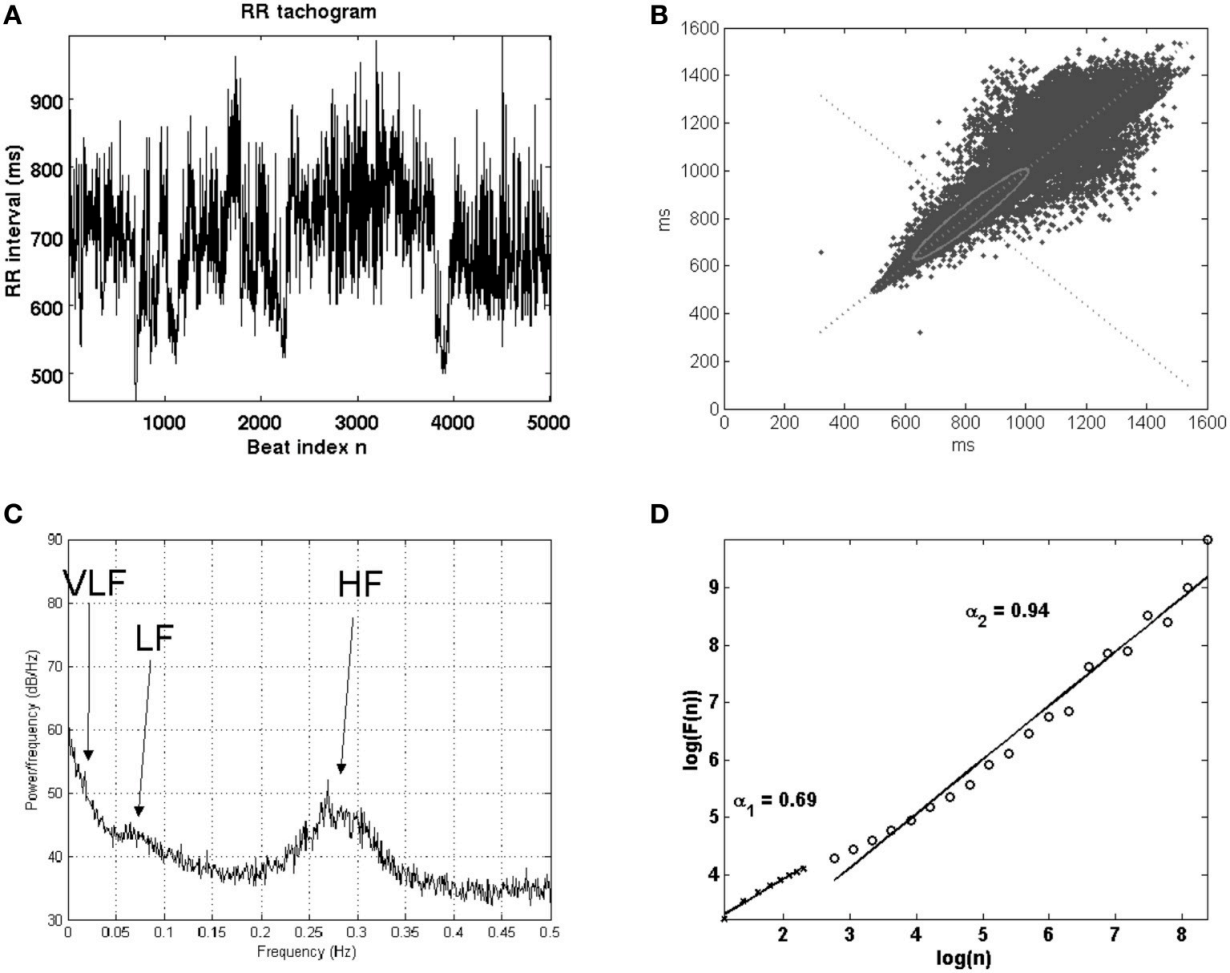
**Example of HRV signal tachogram, or signal given by the times elapsed between consecutive beats as a function of the beat number (A), and the analysis with Lorentz plot (B), power spectral density (C), and nonlinear techniques in terms of detrended fluctuation analysis (D)**.

HRV measures and analyzes the temporal variation between sets of consecutive cardiac beats. The short-term HRV is associated with an adequate control of the heart rate by the autonomic nervous system, whereas the long-term HRV has a more complicated physiological meaning in terms of the complexity of the auto-regulation mechanisms in the cardiovascular system. The autonomic nervous system is divided into two branches, namely the sympathetic and parasympathetic (or vagal). In general terms, the excitation from the sympathetic (parasympathetic) branch is accepted to accelerate (decelerate) the heart rhythm, and since both systems act simultaneously, oscillations about the mean heart rate are produced in a dynamical equilibrium, which can be observed in through the HRV dynamics (Malik et al., 1996). However, this is not the only source of HRV, as far as the nervous system receives information from many other different systems and acting on organs (heart, digestive system, kidney, respiratory system, and more), which also contribute to modulate the HR through a complex dynamic equilibrium with cardiovascular system mechanisms happening in the short- and long-term time scales. Figure 2 shows an example of a HRV signal.

HRV is probably the mostly analyzed index in the cardiovascular risk stratification technical literature, and an important number of models and methods have been developed for this purpose. The European Society of Cardiology and American Heart Association made a first attempt to organize this large amount of initiatives in the field of HRV, trying to propose a set of methodological standards (Malik et al., 1996), which however were limited to a set of recommendations for the compiled signal processing methods to the date. Nevertheless, the large number of techniques for HRV analysis are often organized into families, including statistical, geometrical, spectral, time-scale, and non-linear algorithms. Only those methods applied to SCD prediction are next briefly described, and the interested reader can find the low-level computational details in (Malik et al., 1996; Hossena et al., 2005; Rajendra Acharya et al., 2006; Bilgin et al., 2009; Colak, 2009; Figuera et al., 2013).

#### 4.2.1. Statistical methods

Among all methods, the less sophisticated and yet the mostly used ones, are the statistical indices. These ones are based on the quantification of the RR range or spread from simple statistical measures, such as the mean, the standard deviation, the *pNN50*, and the *HRV index*. The first three mentioned have been paid major attention in the medical literature, whereas the last one is the only statistical index providing a robust behavior against artifacts (Acharya et al., 2007). In practical terms, and very roughly speaking, the rationale behind these methods is the greater HRV, the healthier cardiovascular system. Some of them target to measure the short-term variation (5 min) and some others the long-term variation (daily) in the averaged RR time intervals. Several sets of statistical indices have been also proposed for calculation from the first difference of the RR-series, showing differences in their behavior and predictive capabilities.

Statistical methods have been widely used in the prognosis of SCD and for neuropathy detection, establishing a relationship between the heart enervation degree and the HRV intensity (Acharya et al., 2007), as well as for the diagnosis of diabetic neuropathy in predicting SCD in postinfarction patients (Malik et al., 1996). The SCD prognosis in postinfarction patients has been proposed by using long–term measures of *SDNN*, *pNN*50, *r* − *MSSD*, *HRVindex*, and *LoadIndex*. Among them, *SDNN* and *HRVindex* are absolute measures of HRV (influenced both by the sympathetic and the parasympathetic branches), whereas *pNN*50, *r* − *MSSD* reflect vagal activity, and the *LoadIndex* provides a method to evaluate medium and long term variability (see Malik et al., 1996; Acharya et al., 2007 for details).

#### 4.2.2. Geometric methods

This set of indices aims to improve the robustness of the HRV measurements in RR tachograms, and for this purpose, they distribute the series of observed RR intervals by following a specific geometric pattern, based on the probability density function of normal RR intervals or their first difference, or on the sampling distribution density of normal RR interval durations. Emerging patterns are then measured and classified in different categories and measuring the range or the geometric figure scatter. For instance, when trying to match a given RR histogram with a triangle pattern shape, the parameters better approximating the histogram provide with a measurement of the scatter by means of the triangle basis. The most usual geometrical methods are the the triangular index, the differential index, and the logarithmic index (see Malik et al., 1996 for further details).

#### 4.2.3. Spectral methods

Spectral methods have the capability of distinguishing in the frequency domain the contribution of the sympathetic and the vagal branches, which are mostly confined in specific bands. There are no formal criteria for establishing the limits of each band, as they must be flexible depending on the application, but there is a standard *de-facto* for short-term recordings (from 3 to 5 min long), usually separating into three bands (Montano et al., 1994), namely, Very Low Frequency (VLF, frequencies below 0.04 Hz), Low Frequency (LF, in 0.04−0.15 Hz), and High Frequency (HF, in 0.15−0.4 Hz). Many indices have been proposed according to the many different spectral analysis techniques, but the *LF*∕*HF* power ratio is probably the most useful among the short-term measurements. The spectral analysis mostly used is the Fast Fourier Transform (FFT) and the periodogram, though many advantages have been claimed for the autoregressive methods (Yan and Zheng, 1995), despite their need for a good choice of the model order [often relied to Akaike information criterion (Akaike, 1981)] and for a set of tests (whiteness, residuals) which often are ignored. From a signal processing point of view, special mention is that the HRV signal is a non–uniformly sampled sequence, requiring either working on the beat–frequency (beatquency) domain (Lisenby and Richardson, 1977), or interpolation, which can distort the HF power estimation content. An elegant representation for the HRV signal, which aims to overcome the tachogram limitations in this setting, is given by the Integral Pulse Frequency Modulator (IPFM) model (Mateo and Laguna, 2000), which has been widely followed subsequently in the technical literature, though it has not been proven to be more effective in the SCD risk stratification medical literature.

#### 4.2.4. Time–frequency and time–scale methods

Spectral methods should be used only for stationary processes, and for this reason, time-frequency methods have been proposed instead from the technical literature for analyzing long recordings and transients. A first approach is given by the spectrogram, a simple extension of the frequency analysis in time segments. A second approach, given by the generalized time frequency methods, provide with a continuous surface corresponding to the time sliding windows applied over time. Depending on the window and the frequency domain transformation, a number of different time-frequency representations have been obtained, including the distributions from Choi–Williams, Margenau–Hill, Page, Smooth Pseudo Wigner–Ville, Wigner–Ville, Modified Spectrogram, among others (Mainardi, 2009). A third approach comes from the so called time–scale methods and the popular wavelet transform. These transforms perform the signal decomposition on a set of functions obtained from the so-called mother wavelet and its expansion-contraction and time displacement, hence we talk about time–scale distributions, although the wavelet scale may be readily related to the frequency. Both the continuous (CWT) and the discrete (DWT) wavelet transforms have been proposed for HRV analysis (Mallat and Zhong, 1992; Rajendra Acharya et al., 2006).

#### 4.2.5. Non-linear methods

Special mention requires the characterization of the RR series from the field of nonlinear dynamics, which has recently gained great interest to study the complexity of the cardiac signals. Specifically, Chaos Theory, together with the characterization of a system fractal dimension and the Hurst exponent techniques, have shown possibilities for direct application in the analysis of HRV recordings. Also, the application of higher order statistics (i.e., the bispectrum) has been proposed for highlighting the degree of nonlinearity of the auto-regulation system affecting the HRV (Toledo et al., 2001; Rajendra Acharya et al., 2006). RR series can be analyzed as a complex signal including a certain random behavior. Chaos Theory and Fractal Analysis describe and quantify the complexity of HRV. Fractal dimension and Hurst exponent are the main methods to quantify the complexity for chaotic signals. The Hurst exponent is a fractal index, and as such, it provides a similarity measurement of the signal for the different scale views. In this case, it is calculated as the regression analysis of the averages of the ratio of maximum dispersion over standard deviation, for different window sizes. Based on the Hurst exponent (H), many studies define the Fractal Dimension as the difference of the Euclidean Dimension and the previously defined H. In our particular case, HRV has been shown to behave as a multi-fractal signal, meaning that presents different patterns of fractal behavior over the time. According to recent research, this multi-fractality character is lower when some heart problems are present (Sassi et al., 2009).

### 4.3. T–wave alternans

**Figure 3:**
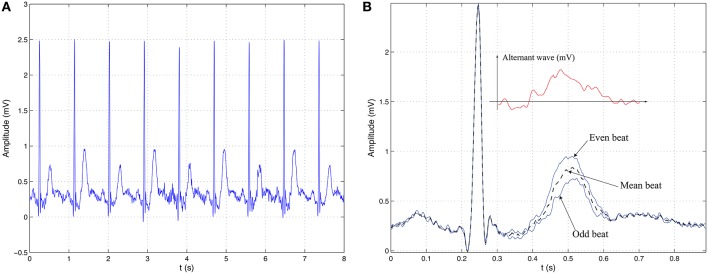
**Illustrative example of TWA. (A)** ECG signal with periodic pattern alternation in the repolarization segment with a period of two beats. **(B)** Visual interpretation of TWA as the difference between the averaged event beat and the averaged odd beat.

TWA is a particular type of ECG alternans that is related to changes in amplitude, waveform, and duration of the ST–T complex occurring on an every–other–beat basis. Also known as repolarization alternans, it has been shown to be a clinical marker for stratifying risk in SCD patients (Walker and Rosenbaum, 2003). Figure 3 depicts an example of a severe TWA where the periodic pattern of two beats is clearly observed on the ECG of the left panel. The case corresponds to an alternant wave of 300 μV. To better identify this variation, even and odd heartbeats can be separated into two subsets to be posteriorly aligned and averaged. The resulting even and odd beats can then be superimposed as in Figure 3B, enhancing the differences between them and making thus easier the visualization of alternans. Also in this plot, but in a separate graph, the estimate of the alternant wave is depicted, which is determined as the difference between the averaged even and odd beats. This phenomenon is generated at the myocites level and it may be caused by differences among action potential shapes. Thus, alternation can be given because different regions have distinct and non-natural action potential durations (modified spatial dispersion of repolarization), or by alternate duration of action potentials of a single cell (temporal dispersion) (Narayan, 2006; Bakhshi et al., 2013).

Ventricular repolarization alternans is usually referred to as microvolt–TWA (MTWA) because in most cases the fluctuations are so small in surface ECG that visual assessment is impractical. In consequence, its manual identification is almost unfeasible and the introduction and use of signal processing techniques is unavoidable for its analysis, detection, and estimation. Despite this unnoticeable manifestation, the physiological and clinical importance of these subtle variations might be explained because at the cellular level the magnitudes may be several orders greater (Walker and Rosenbaum, 2003).

Alternans depend on heart rate, which is a modulating factor for TWA, i.e., the alternans magnitude increases at increasing heart rates. In fact, ventricular repolarization alternations can be very well found at higher cardiac frequencies in healthy individuals, yet the onset heart rate for TWA is considerably lower in patients at risk. Thus, during atrial pacing to predict induction of ventricular tachyarrhythmias, the onset heart rate was found to be 95±15 bpm, and the specificity at 120 bpm was very low, i.e., amounts of alternans were detected in control group at 120 bpm. It was fount that the most suitable cardiac rhythms for predicting were under 110 bpm (Tanno et al., 2000, 2004). In summary, in common practice, heart rate must be elevated to pass a TWA test for assessing cardiac risk and it has to be done with care and under supervision. Atrial pacing during EPS is one means to increase cardiac frequency, but other methods less harmful and noninvasive, such as exercise testing, are currently more extensively used because they have shown comparable results (Hohnloser et al., 1997).

Although most clinical studies have relied on this heart rate dependency procedure to find predictive TWA, the analysis in ambulatory ECG records has yielded promising results (Verrier et al., 2011). Therefore, the use of this type of data, which avoids the exercise–based test described above, is opening a new perspective of great interest toward a more versatile way of testing with less intervention from clinicians and in a more convenient fashion for patients.

So far, plenty of methods to automatically tracking TWA have been reported due to the strong evidences that tie ventricular repolarization alternans with risk stratification. However, the difficulty of visually identifying alternans has prevented the design of specific annotated databases, and consequently, the definition of a gold standard to accomplish the performance validation of the proposed methods. Therefore, from a clinical standpoint, there are to date only two methods that have demonstrated their validity because they have been employed in a number of clinical studies (Verrier et al., 2011): the Spectral Method (SM) (Rosenbaum et al., 1996) and the Modified Moving Average (MMA) (Nearing and Verrier, 2002). For these reasons, and also for the sake of simplicity and their illustrative capacity, we center our attention on them to introduce the basics of alternans detection and estimation. Both methods are briefly explained in their simplest version.

In finding TWA, the attention is driven to the ventricular repolarization segments of the ECG with the purpose of characterizing a periodic pattern every–other–beats. Thus, the set of *N* samples from the *m*–th ST–T complex can be enclosed into the *N* dimensional vector

(2) $$\begin{array}{l} {\text{x}}_{m}={\left[x_{m}\left(0\right),x_{m}\left(1\right),\cdots \;,x_{m}\left(N-1\right)\right]}^{T} \end{array}$$

We then row–wise allocate the repolarization segments into the *M* × *N* matrix:

(3) $$\begin{array}{l} \text{M}={\left[{\text{x}}_{0}^{T},{\text{x}}_{1}^{T},\cdots \;,{\text{x}}_{M-1}^{T}\right]}^{T}=\left[{\text{s}}_{0},{\text{s}}_{1},\cdots \;,{\text{s}}_{N-1}\right] \end{array}$$

so as to gather *M* consecutive beats for testing. Column–wise, the *M*×1 vector ${\text{s}}_{n}={\left[s_{n}\left(0\right),s_{n}\left(1\right),\cdots \;,s_{n}\left(M-1\right)\right]}^{T}$ contains the samples of *M* consecutive heartbeats collected at the same time latency *n*. This sequence is known as a beat series.

When analyzing TWA in surface ECG, the signal coexists with severe level of noise and artifacts due to exercising or ambulatory recording. As this undesirable component interferes with a correct interpretation, many different preprocessing blocks for signal conditioning are considered, such as linear filtering, baseline wander elimination, QRS detection and delineation, beat alignment and rejection, among others (Martínez and Olmos, 2005). Matrix **M** in Equation(3) can be seen as the outcome of several of these preprocessing stages. The design of these blocks is accomplished from different viewpoints and according to many different approaches.

The SM identifies TWA in the spectral domain of the beat series, also referred to as beatquency domain. Thus, the periodic pattern of 2, which for existing TWA is inherent in each column of **M**, is reflected as the component at 0.5 cycles/beat (repetition every two beats). Thus, the SM estimates the power spectrum density function of each beat series through the periodogram:

(4) $$\begin{array}{l} P_{n}(f)={\left|\frac{1}{M}\overset{M-1}{\underset{m=0}{\sum^{\text{​}}}}s_{n}(m)e^{-j2\pi fn}\right|}^{2} \end{array}$$

for *n* = 0, 1, ⋯ , *N*−1. These spectra, generated at each point of the repolarization segment, are averaged to gather the contribution of the whole ST–T segment into an aggregate spectrum :

(5) $$\begin{array}{l} P\left(f\right)=\frac{1}{N}\overset{N-1}{\underset{n=0}{\sum }}P_{n}\left(f\right) \end{array}$$

The *K* score, also known as TWA ratio, determines the magnitude of the power spectrum at the alternans frequency over the noise:

(6) $$\begin{array}{l} K=\frac{P\left(0.5\right)-\mu_{noise}}{\sigma_{noise}} \end{array}$$

where *P*(0.5) is the value of the aggregate spectrum (Equation 5) at 0.5 cycles/beat, and μ_*noise*_ and σ_*noise*_ are the mean and the standard deviation of noise, which is estimated in an adjacent reference band close to the alternans frequency, typically around 0.4 cycles/beat. A *K* score is taken as statistically significant when the alternans component exceeds three times the level of noise, i.e., *K* > 3. The estimate of the alternant wave is determined as

(7) $$\begin{array}{l} V_{alt}=\sqrt{P\left(0.5\right)-\mu_{noise}} \end{array}$$

which represents the mean magnitude of the difference between the amplitude of an even or odd beat with respect to the mean beat (Rosenbaum et al., 1996). As the SM is based on spectral analysis, it requires quasi–stationary conditions during long period of times. Typically, the number of heartbeats to obtain an aggregate spectrum is set to be *M* = 128. At the same time during exercise, the patient is suitably guided so that its heart rate can be raised up to the valid range.

The MMA is a different approach that relies on nonlinear averaging to estimate an alternant wave. This method operates in the time domain to determine even and odd ST–T segment estimates as follows:

(8) $$\begin{array}{l} {\hat{\text{x}}}_{m}={\hat{\text{x}}}_{m-2}+{\text{h}}_{m-2} \end{array}$$

where *m* = 2, 3, ⋯ , *M*. Notice that the MMA works over separated even and odd beats. The variable ${\hat{\text{x}}}_{m}$ stands for the estimate of **x**_*m*_ and at the initial instance: ${\hat{\text{x}}}_{0}={\text{x}}_{0}$ and ${\hat{\text{x}}}_{1}={\text{x}}_{1}$. The array **h**_*m*_ is a correcting factor that depends on a fraction of the error estimate

(9) $$\begin{array}{l} {\text{e}}_{m}=\frac{{\text{x}}_{m}-{\hat{\text{x}}}_{m}}{8} \end{array}$$

Each entry of **h**_*m*_ is determined as follows:

(10) $$\begin{array}{l} h_{m}(n)=\{\begin{array}{cc} -32, & e_{m}(n)⩽-32\\ e_{m}(n), & -32<e_{m}(n)⩽-1\\ -1, & -1<e_{m}(n)<0\\ 0, & e_{m}(n)=0\\ 1, & 0<e_{m}(n)⩽1\\ e_{m}(n), & 1<e_{m}(n)⩽32\\ 32, & e_{m}(n)⩾32 \end{array} \end{array}$$

where *e*_*m*_(*n*) is the *n*–th element of **e**_*m*_ and *n* = 0, ⋯ , *N* − 1. Whit this simple processing, a measure of the alternant wave is achieved as the difference between even and odd estimates

(11) $$\begin{array}{l} {\text{v}}_{l}={\hat{\text{x}}}_{2l-1}-{\hat{\text{x}}}_{2l-2} \end{array}$$

where $l=1,\cdots ,\left\lfloor \frac{M}{2}\right\rfloor$, and the TWA detection parameter, referred to as maximum alternans magnitude, is computed as the maximum of the absolute value

(12) $$V_{l}=\underset{n}{\max}\left|{\text{v}}_{l}\right|$$

The MMA can be well applied during exercise testing because it has some noise reduction capacity due to the nonlinear limit function in Equation (10), and it can report an outcome with less delay than that of SM. Typically, the TWA level is reported every 10–15 s (Nearing and Verrier, 2002), making the MMA more versatile and more appropriate if we want to deal with ambulatory recordings and hence avoid the exercise tests. Risk stratification is analyzed by means of the maximum alternans magnitude, *V*_*l*_, though the exact cutpoint has not been defined yet. Thus, values such as *V*_*l*_ ≥ 60 μV and ≥ 47 μV have been associated with high level of SCD risk (Nearing and Verrier, 2002; Verrier et al., 2011).

TWA is a very well defined signal processing problem, and this is another encouraging reason to address this matter from different viewpoints. Among them, we can highlight the following methods, which are described within an unified framework in Martínez and Olmos (2005): the Complex Demodulation method (Nearing and Verrier, 1993); the Correlation Method (Burattini et al., 1999); a method based on the Karhunen–Love Transform (Laguna et al., 1999) and Capon filtering (Martínez et al., 2000); methods using the Poincar mapping (Strumillo and Ruta, 2002), the periodicity transform (Srikanth et al., 2002a), statistical *t*–test (Srikanth et al., 2002b), and the Laplacian Likelihood Ratio (Martínez et al., 2006). Recently, matched filtering (Bashir et al., 2014) and wavelet transform (Romero et al., 2008) have been used in this issue. As well, the subject of TWA has been tackled from a multilead perspective (Monasterio et al., 2010), and other approaches are aimed at improving the performance by introducing additional preprocessing blocks or modification of existing methods (Cuesta-Frau et al., 2009; Blanco-Velasco et al., 2010; Ghoraani et al., 2011; Nemati et al., 2011). Regarding the preprocessing blocks, it is well known that a correct choice influences the behavior of posterior stages. However, there is no consensus about the design of these systems, as well as the need of their presence. Given that the final detection-estimation performance can be significantly affected for the choice of a preprocessing blocks, the study of the impact of these parts to the whole detector deserves specific attention (Goya-Esteban et al., 2014).

## 5. Technology transfer

The analysis of technology transfer is not an easy task. In this work, we restrict ourselves to a compilation on the number of patents related with each of the kind of computational indices in the preceding section, and a comparison with examples of the algorithms used in commercial systems. It has to be kept in mind that the interpretation of the number of patents as technology transfer can be controversial, as far as it can be more related with the expectations in the commercial field and even with fashion in the research and development of the electro-medicine field. Nevertheless, we consider that interesting conclusions can be obtained from this analysis.

### 5.1. HRT computational indices

The technology transfer in the HRT environment can be explored from two different points of view, namely, the patents that have been generated from this concept, and the use in medical systems and devices.

The original proposal for HRT was patented by Schmidt (2002), as a method for evaluating patients with successive heartbeats directly before and after a VPB, while using the time intervals from the sequence before as a reference value. The patented method is claimed for comprising two steps: (a) determining characteristic attributes of heartbeats occurring in a continuous sequence immediately preceding and following the VPB; and (b) quantifying these attributes using an analysis method to produce a result, to be compared to a reference value.

Other patents have followed disclosing technology related to HRT, but mostly focused on their use in ICD. A representative example is delivered in (Kornet and Schneider, 2011), where a set of techniques is proposed for predicting the occurrence of an arrhythmia based on HRT indications, in the domain of medical devices sensing electrical signals within a patient. Accordingly, a medical device may identify abnormal heartbeats and measure HRT resulting from them, or even providing one or more pacing pulses to the heart, and when the measured HRT related parameter deviates from a baseline, the medical device may predict the arrhythmia occurrence. HRT measurements may be derived based on heart rate, e.g., from an EGM or ECG, but also from pressure, impedance, movement, sound, flow, optic, or chemical signals. The medical device may provide a therapy configured to prevent the predicted arrhythmia from occurring, reduce an effect of the arrhythmia, or terminate the arrhythmia.

In Farazi (2011), a method and apparatus for using vagal stimulation to detect autonomic tone and assess a patients risk of SCD are presented, involving the stimulation of the patients vagus nerve in order to induce a drop in arterial blood pressure, hence simulating the patients cardiovascular response to a VPB. Sinus rhythm just before and after the stimulation is recorded and analyzed. In an embodiment, the method is implemented in an ICD, which can deliver arrhythmia prevention therapy based on the risk of SCD. In Burnes et al. (2006), a similar device is envisioned being capable of gauging hemodynamic status, yielding any issues as quickly and appropriately treated, as well as providing diagnosis for hemodynamic status within an implantable device, or providing a criterion for optimizing the performance of a pacemaker. The implanted device determines the hemodynamic status of the patient by observing the perturbation in the heart rate (natural or stimulated), measuring HRT and quantifying it. In Messier et al. (2013), sets of techniques are disclosed for generating a risk stratification indicator based on HRT measurements in ICD, among them the HRT measurements.

With respect to implementations, the MARS system from GE for Holter analysis (GE, 2005) uses the method originally proposed by Schmidt for ECG, including the TO, the TS, and the turbulence correlation (i.e., the steepest correlation coefficient of the linear regression through five consecutive measurements in the averaged tachogram). This implementation is strongly supported by scientific evidence in (Schmidt et al., 1999; Watanabe et al., 2002; Barthel et al., 2003). The system consists of an analysis program for ECG signals, providing measurements of HRT in patients undergoing cardiovascular disease testing for interpretation by qualified health care practitioners for the purposes of risk stratification and prediction of SCD. The HRT analysis program is stated to only provide measurements, not interpretations, and to be used in conjunction with the patient's clinical history, symptoms, and other diagnostic tests for final clinical judgment.

### 5.2. HRV computational indices

**Figure 4:**
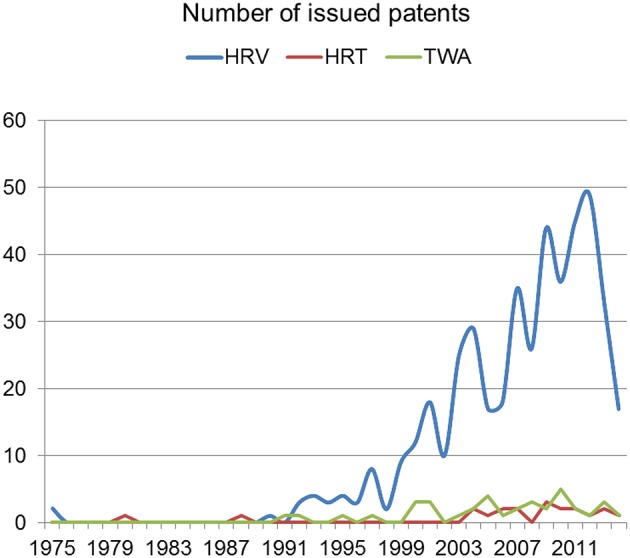
**Number of issued patents by year at European Patent Office (adapted from Espacenet)**.

Several thousands of patents referring to HRV can be found, where the HRV methods are implicit in a given specific device or defined as a method for different identifications and measurements. Just on a Google patent search, about 12.000 entries can be found containing the keyword HRV, and then no surprisingly a few dozens of them are related to SCD, were almost 80% of them were issued after 2005. According to Espacenet database of European Patent Office, 325 patents are related to HRV, and following the same intensification as the woldwide patents, this effort that is being enhance in recent years (see Figure 4). Information found in Scopus, Espacenet, and Google suggests that the same shape in terms of intensification in the last 3–7 years is coincident in the patents and publications with regards to HRV to predict or analyze SCD, although as far as existing commercial devices is concerned, virtually none of them are effectively providing scientific evidence to be used from a clinical point of view.

There is a variety of software for free distribution that supports the analysis of HRV in its different modalities. Physionet repository (Goldberger et al., 2000) conveys most of signal processing proposed indices in the literature, and is a world-wide domain in this setting. On the other hand, Kubios-HRV is a free software allowing to perform HRV analysis, including a variety of time-domain, frequency-domain and nonlinear analysis methods (Tarvainen et al., 2014). Up to 2014, it had over 16,000 downloads, and it has become a very popular tool, specially in sports applications.

With respect to software implementation in commercial systems, a wide variety of them can be found. For instance, GE-HRV (GE, 2005) offers a software program for the measurement of HRV in conjunction with the MARS Ambulatory ECG System. GE system performs an interpolation to obtain clean an equally spaced sequences, and provides with frequency-domain indices based on the periodogram spectrum estimation, using a 1024 point FFT and a Hanning window. As another example, Mortara (Mortara Instrument Inc., 2008) offers an HRV module in the H-Scribe system, which is a complete Holter ECG analysis package allowing the user to analyze Holter recordings, and it incorporates several well-known HRV time-domain indices, namely, *pRR*50, RMSSD, or SDNN. As a final example, Sorin SyneScope (Sorin Group, 2007) is a Holter ECG package which allows the user to analyze Holter recordings, and it can be extended with a specific HRV analysis package. This package allows to remove ectopic beats and artifacts by using linear interpolation, and it computes usual time-domain and frequency-domain HRV measurements.

### 5.3. TWA computational indices

Currently, the feature that mainly hinders the development and transfer of novel and powerful methods for TWA characterization is the lack of a gold standard. Therefore, the assumption of this type of technology relies on the number of developed clinical prospective studies as the means to testify scientific evidence. So far, most of this prospective studies have been conducted with the SM (Verrier et al., 2011), and for this reason is the most widely accepted analysis. Thus, this method (originally patented in Cohen and Smith, 1989) is used by Cambridge Heart, Inc. in several of their products such as HearTwave II and CH2000. The procedure here is applied under ergometry, either in a treadmill or bicycle ergometry, and supervised by trained professionals. Although the effort level during exercise is mild and just enough to attain the target heart rate for finding pathological TWA, the amount of noise and artifact is significant, so the method requires additional special electrodes to reduce these non-desirable components. As the SM method has been utilized in many clinical studies, it has been accepted by Medicare in USA for reimbursement.

The MMA method has also been fairly tested in clinical studies, although in less occasions than the SM. Due to its robustness and nice time domain achievement, it is enclosed within the Marquette analysis programs by GE for ECG processing, and it is applied in two different products. On the one hand, given its capability to find TWA in ambulatory ECG, it is implemented in the ambulatory MARS system, but as it can also interpret alternans during stress testing, it is also working on the Case Assessment System for Exercise Testing (CASE).

Beyond the methods that have participated in large clinical prospective studies, Sorin Group includes in its Holter system, SyneScope, a feature for the analysis of the T wave variability based on the patent (Couderc and Zabera, 2007). It is a time domain method aimed at detecting repolarization alternans. It works over beat series obtained as a sequence of samples corresponding each one to a single value per beat, which could be, for instance, the mean values of a portion of the ST–T segments. The variability is assessed by means of an index whose computation is based on the analysis of alternation changes of the referred beat series.

## 6. Scientific evidence

After we have revised technical and technological considerations on the use of ECG based indices for SCD risk stratification, it is useful to address now, with all the previous information in mind, the current status of the clinical viewpoint in terms of their application in practice. Whereas, a number of clinical studies exist on the subject, and deep clinical reviews are available in the medical literature, this section aims to give a landscape on several of the main trials in the field, their relationship with the technical considerations, and the points to be taken into account for a better understanding on the current state of art in the signal processing setting.

### 6.1. Patients with coronary artery disease

The topic of SCD risk stratification started with Ischemic Cardiopathy patients. In the 70s, patients with usual VPB and (or) NSVT in Holter were associated to higher mortality, specially when associated to ventricular disfunction. In the 80s and 90s, the study of the autonomic factors was intensely scrutinized, and a number of studies were made on the predictive value of HRV, together with other non-invasive parameters (see e.g., Farrell et al., 1991), with St. George's Hospital in London being probably the most relevant contributing group by then.

Initially encouraging results turned complicated later, due to several different reasons. First, many new spectral indices of HRV were proposed, from both spectral and non-spectral domains, which made complicated to make comparisons among series. In addition, HRV was dependent on many factors (age, diabetes, drugs) which also made data difficult to interpret. Second, it was established that treatment with beta-blockers was an essential approach in post-infarction patients, hence making measurements on the autonomic tone in beta-blocked patients was a contradiction. Third, St. George's group suggested that the averaged heart rate as such had a value which was comparable to HRV, hence the search for sophisticated HRV indices could be questioned (Copie et al., 1996). And fourth, many groups started to publish many other measurements on the autonomic tone, such as HRT, response to drug infusion, heart rate acceleration and deceleration, oscillations, or changes in repolarization. This made complicated to put all the pieces together and created a maze of knowledge in this setting, from a clinical point of view. The ATRAMI study was designed aiming to clarify the field (La Rovere et al., 1998), and it showed the independent predictive value of autonomic indices. However, the study presented many limitations, as far as important variables which were not included in the multivariate analysis (Barron and Viskin, 1998), and also, one of the methods that were used for the autonomic evaluation was adrenaline perfusion, which is an uncomfortable test. The study was not convincing, hence it reached no practical impact.

In the meantime, there were changes in handling patients with myocardial infarction, including the early revascularization, the use of beta-blockers and of angiotensin converter enzime inhibitors, so that the clinical profile of post-infarction patient had changed. The new reevaluation of classical risk factors (LVEF, frequent VPB in Holter, NSVT in Holter) and of HRV indices in this modified population with the new treatments showed the LVEF being the determinant factor. One of the main scientific supporters of the role of the nervous autonomous system in the post-infarction mortality risk, who participated in ATRAMI trial, admitted that: *In the modified situation created by thrombolysis and advanced therapeutic regimens, depressed LV function is the only risk stratifier that has not lost its predictive value. Ongoing primary prevention trials with ICDs and mortality trials with antiarrhythmic drugs randomize mainly on the basis of EF, and there is growing evidence for accentuated benefit of ICDs among patients with greater impairment of systolic function* (La Rovere et al., 2001).

On the other hand, LVEF is easy to obtain (it just needs an ecography), it is a routinely used parameter by the cardiologist, and it is universally obtained in patients with anterior myocardial infarction (AMI). On the contrary, parameters based on nervous autonomous system activity require a 24 h Holter (not fully comfortable, and not always well accepted by the patient), which has to be be carefully read and annotated (between 20 and 30 m of time from a cardiologist), and a specific HRV analysis software (until recently, not available in all current Holter systems). Moreover, it can not be used in a number of patients (including atrial fibrillation, pacemakers, or frequent VPB), and cut-off values and parameters are unclear and probably change with age, treatment, and other factors. Then, it seems natural that the big multicenter trials first trying to show that ICD implantation in primary prevention (i.e., in patients with no documented arrhythmia to that moment) reduced the mortality, were designed according to the classical factor of EF. Several relevant trials were driven, including MADIT I (1996), MUSTT (1999), MADIT II (2002), and SCD-Heft (2005), all of them showing significant mortality reduction with ICD and all of them using LVEF as inclusion criterion, so these studies have defined the clinical practice until now. In two additional studies, other inclusion criteria for the study were applied in addition to LVEF, namely, late potentials in Cabg-Patch and a HRV parameter in DINAMIT, both with negative results. Both studies had limitations to this respect, and other factors to be taken into account, but they contributed to the idea that LVEF is the valid approach for SCD risk stratification and ICD indication.

In this scenario, risk stratification of post-AMI SCD with autonomic parameters derived from 24 h Holter is seldom used in the clinical practice. It seems evident that patients with depressed LVEF have bad prognosis and they need an implanted ICD, and it seems unlikely that the analysis of the autonomous function will gain interest in this group. Greater interest can be present in the patients with relatively well-conserved LVEF (>0.35), in which there is low incidence of SCD, but in turn it represents a much larger population, specially with current AMI treatment that are capable of avoiding the severe ventricular disfunction in most of patients. If it were possible to find a high-risk subgroup with other criteria within the well-prognosis group according to LVEF, then the ECG based indices techniques could be extremely useful. This is the direction followed by trials like REFINE (Exner et al., 2007a), which included patients with LVEF < 0.50 and measured HRT and TWA, and like ISAR-Risk (Bauer et al., 2009), which used HRT and deceleration capacity. Both of them obtained interesting results in terms of SCD risk prediction with these variables, and for these results to be adopted by the clinical practice, a study randomizing high SCD risk patients to ICD vs. non-ICD should be required. An effort in standardization of existing techniques should also be required, as their inclusion in conventional Holter systems, and pruning of variables that can lead to confusion, among others.

### 6.2. Non-ischemic dilated myocardiopathy and others

In non-ischemic dilated myocardiopathy (NDM) patients, the only study showing ICD efficacy for preventing the mortality was the SCD-Heft, and it was based on the LVEF. Moreover, the largest study in NDM to evaluate the predictive value of other non-invasive parameters (including HRV, HRT, T wave micro-alternans, and late potentials), was the Marburg trial, and it yielded negative results, as LVEF was the only predictive factor for ventricular arrhythmias (Grimm et al., 2003). After these precedents, no big-scale trial has been subsequently designed with other potentially predictive factors derived from the autonomous nervous system or from the TWA.

Finally, there are no long series reported in other cardiac pathologies with SCD risk (such as the hypertrophic myocardiopathy, the Brugada syndrome, or the right ventricular arrhythmogenic dysplasia), and the few available research suggests that these pathologies do not significantly modify the HRV parameters, and that these alterations have no clear prognostic value. On the other hand, these patients have few VPB, so the HRT cannot be estimated in a number of them. In this setting, the SCD risk stratification depends on specific factors for each pathology, which are radically different from those ones used in post-AMI and in NDM.

### 6.3. On scientific evidence for TWA

The TWA analysis has many disadvantages shared with the autonomous nervous system analysis. It is not easy to measure, and it is recommended to analyze with increased heart rate, which requires a stress test (to be performed under ergometry, unavailable in many centers, and it requires the cardiac patient to be capable of exercising). Other options are cathecolamins infusion (long and uncomfortable procedure) or atrial stimulation (invasive procedure). However, an expensive additional tool is required, with special electrodes (low-noise designed) and complex to handle and to interpret. As a consequence, it is not widely extended. The option of measuring TWA from the Holter is also complicated, it is not fully validated, and it requires specific software. In both cases, this set of indices are not useful in patients who are not in sinus rhythm, and it is not clear how to manage beta blockers if they are being administered.

Though several methods have been described for measuring TWA, their cut-off values are not yet well established. Some series have provided with positive results for this technique, such as ABCD trial (Costantini et al., 2009), and others with negative results, such as MASTER trial (Chow et al., 2008). There has been no random study to date on ICD implantation suitability as a function of the results of TWA indices. The ABCD trial was somehow close to this approach, but it compared TWA with invasive EPS, which is used only in some groups today, and the ICD is implanted in most of patients in this group of assumed low risk. Hence, these results are not much applicable in practice. In summary, this technique seems to be still far from its use in the clinical practice. It should be helpful to have a simple and inexpensive estimation technique, as well as a clearer standardization. These advances would allow us to have clinical results more quickly, to extend its use to a larger number of centers, and in the middle term, to design an international randomized and powerful study showing its actual usefulness.

## 7. Discussion and conclusions

The computational techniques used for measuring ECG derived markers of SCD are quite diverse. A qualitative summary of several processing methods for a selected set of representative indices has been presented. In this setting, the scientific evidence, as given by the conduction of populational clinical studies in representative patients databases, is not always strong enough to support the different proposed algorithms. The limitations of many of the previous studies do not allow their reliable generalization, so that the ability to discriminate patients at SCD risk is still far from satisfactory. The fluid technology transfer from scientific evidence to practical applications also remains a pending issue in the field. Some of the scientific evidences already present in literature, as well as some patents recently issued, do not correspond to the state of the art in clinical applications, but instead they can be seen as belonging to the future of the technology. This rationale motivates the evaluation of recent findings, together with a prospective open approach, in seeking for new indices and methods.

Current data gathering techniques allow the analysis of different types of risk markers to determine whether a patient may suffer sudden death. However, the causes of SCD come from many ways and may be due to different mechanisms, so the current cardiac risk markers cannot be arbitrarily applied to any situation. Basically, predicting sudden death is strongly dependent on the risk group to be analyzed. As a simple example, SCD prediction in general population is different than prognosing long QT patients, and neither of them has to do with hypertrophic myocardiopathy or myocardial infarction. Thus, every group has their own useful predictors, e.g., HRV may be valuable in infarction, but not for channelopathies. In summary, we may not assert that one specific marker can be used in general to stratify cardiac risk due to the aforementioned reasons.

Many techniques for SCD risk stratification have been proposed to date, but according to the clinical studies that have been carried out, they still show limited capabilities mainly due to their poor sensitivity and positive predictive value (Exner et al., 2007b; Goldberger et al., 2008; Kreuz et al., 2008). In general, there is no universal marker to predict SCD and the best choice may strongly depend on the pathology under study. Moreover, clinical markers arise from many different sources (ECG, echocardiogram, blood analysis), which are more or less informative depending on the specific cardiac disease group. Therefore, an approach for studying these cardiac markers should take into account the way they are collected, but special attention must be driven to the origin and causes of SCD.

Finally, we only have transversal data from HRT, HRV, and TWA, in the sense that we measure them one day and we analyze whether they have predictive value in the middle or long term. With new technologies allowing repetitive and long-term monitoring, it seems possible to have longitudinal data and include the time evolution of these parameters in the stratification algorithm, its usefulness being pending of the future research. New scenarios like electronic health recordings, big data, long-term monitoring, and cloud databases, could provide with suitable new paradigms in the near future.

## Author contributions

FG: Conception of the Review, writing, and proof-reading for each section in the paper. MB: Conception of the Review, writing, and proof-reading for each section in the paper. OB: Contribution to the Heart Rate Turbulence and Heart Rate Variability sections, writing, and proof-reading. AG: Conception of the Review. Contributions in each of the section, writing, and proof-reading for each section in the paper. JR: Conception of the Review. Contributions in each of the section, writing, and proof-reading for each section in the paper.

### Conflict of interest statement

The authors declare that the research was conducted in the absence of any commercial or financial relationships that could be construed as a potential conflict of interest.
